# Inhibition of angiogenesis by platelets in systemic sclerosis patients

**DOI:** 10.1186/s13075-015-0848-2

**Published:** 2015-11-19

**Authors:** Daniela Hirigoyen, Paula I. Burgos, Veronica Mezzano, Josefina Duran, Magaly Barrientos, Claudia G. Saez, Olga Panes, Diego Mezzano, Mirentxu Iruretagoyena

**Affiliations:** Departamento de Inmunología Clínica y Reumatología, Pontificia Universidad Católica de Chile, Marcoleta 350, Santiago, Chile; Departamento de Hematología-Oncología, Facultad de Medicina, Pontificia Universidad Católica de Chile, Santiago, Chile

**Keywords:** Systemic sclerosis, Platelets, Vascular endothelial growth factor, Transforming growth factor β

## Abstract

**Introduction:**

Systemic sclerosis (SSc) is a chronic autoimmune disease characterized by microvascular damage, inflammation, and fibrosis. It has become increasingly evident that platelets, beyond regulating hemostasis, are important in inflammation and innate immunity. Platelets may be an important source of proinflammatory and profibrotic cytokines in the vascular microenvironment. In this study, we sought to assess the contribution of platelet-derived factors in patients with SSc to the angiogenesis of human dermal microvascular endothelial cells (DMVECs) in a tubule formation assay and to characterize the secretion of profibrotic and proinflammatory cytokines in these platelets.

**Methods:**

We analyzed platelets obtained from 30 patients with SSc and 12 healthy control subjects. Angiogenesis was evaluated in vitro with a DMVEC tubule formation assay on Matrigel and platelet-derived angiogenic factors such as vascular endothelial growth factor (VEGF), 165b isoform (VEGF_165_b), and cytokine secretion was evaluated. Platelet serotonin content was also determined.

**Results:**

When DMVECs were incubated with SSc platelet releasates, tubule formation was significantly inhibited (*p* < 0.01, *t* test), and higher expression of endothelin-1 in these cells was observed compared with control subjects (*p* < 0.05, Mann–Whitney *U* test). In SSc platelet releasates, VEGF_165_b was significantly higher (*p* < 0.05, *t* test), and the VEGF_165_b/VEGF ratio was increased compared with that of control subjects. Higher secretion of transforming growth factor β (*p* < 0.01, *t* test) and CD40L (*p* < 0.01, *t* test) was observed compared with control subjects. Also, intraplatelet serotonin levels were lower in platelets obtained from patients with diffuse SSc compared with patients with limited SSc and control subjects (*p* < 0.05, *t* test).

**Conclusions:**

Our findings suggest that antiangiogenic factors such as VEGF_165_b, together with proinflammatory and profibrotic factors secreted by platelets, can contribute to the progression of peripheral microvascular damage, defective vascular repair, and fibrosis in patients with SSc.

## Introduction

Systemic sclerosis (SSc), or scleroderma, is a chronic autoimmune disease characterized by vasculopathy, immune deregulation, and fibrosis of the skin and internal organs. It has an estimated prevalence of about 1–2 per 100,000 persons [1]. Women are at much higher risk for developing scleroderma, generally starting with symptoms in middle age [2]. Clinically, the disease is classified into two major subgroups according to the extension of skin involvement (limited or diffuse cutaneous scleroderma) and the autoantibody profile. Both types can present with vascular involvement, Raynaud’s phenomenon and obliterative vasculopathy, and systemic fibrosis [3]. The pathogenic triad involving vascular injury, autoimmunity, and fibrosis (scleroderma trifecta) is classically considered a hallmark of the disease, but the interplay among these distinct processes, which initiate and sustain the progressive tissue damage in scleroderma, requires further exploration [4–6]. Both autoimmunity and vasculopathy appear to precede the onset of the disease, and contribute to the progression of fibrosis.

Platelets play key roles in hemostasis, thrombosis, and tissue repair and, as more recently described, in inflammation and innate immunity [7]. Platelets are currently being used, among others, for regenerative treatments after bone surgery and cosmetic surgery, although solid evidence of their regulatory functions is missing. Endothelial dysfunction and immune regulatory mechanisms may be associated with platelet function. It has been proposed that platelets can play a role in SSc because enhanced activation and an increased tendency to aggregation have been described in these patients [8].

Platelets could substantially contribute to the pathophysiology of scleroderma through several mechanisms. The levels of several platelet-derived molecules, such as β-thrombomodulin and platelet-derived growth factor, are elevated in the serum of patients with SSc [8, 9]. In addition, elevated levels of circulating platelet aggregates have been described [8]. Silveri et al. found higher levels of endothelium-derived endothelin-1, tissue type plasminogen activator, plasminogen activator inhibitor type 1, and platelet-derived growth factor in patients with Raynaud’s phenomenon associated with scleroderma than in control subjects, suggesting platelet activation in vivo and endothelial damage [10]. However, the exact contribution of platelets and platelet-derived factors in the pathogenesis of SSc and other fibrotic diseases has not yet been established.

Our hypothesis is that platelets contribute to maintaining the microvascular injury that leads to fibrosis in patients with SSc. The aim of this study was to further characterize the role of platelets in vascular damage in patients with scleroderma and their proinflammatory and profibrotic cytokine profiles. In particular, the angiogenesis of human dermal microvascular endothelial cells (DMVECs) was evaluated in the presence of platelet supernatants obtained from patients with SSc. Also, antiangiogenic factors such as vascular endothelial growth factor (VEGF), 165b isoform (VEGF_165_b), were assessed. Serotonin content, cytokine secretion profile, and fibroblast proliferation and stimulation by SSc and control platelets were also evaluated. Our results add further evidence for the participation of platelets in microvascular damage and fibrosis induction in SSc.

## Material and methods

### Patients

We studied platelets derived from 30 patients with SSc, including both women and men (mean 51.3 years), and from 12 healthy control subjects. The diagnosis of SSc was made according to the American College of Rheumatology criteria [11], and the patients were classified into diffuse and limited types according to the criteria described by Le Roy et al. [12]. The clinical characteristics of the patients are summarized in Table 1. All samples were obtained after the patients and control subjects gave their written informed consent in accordance with the Declaration of Helsinki. The ethics committees of the Pontificia Universidad Católica de Chile and Comisión Nacional de Investigación Científica y Tecnológica (CONICYT) approved the entire study protocol. Patients and control subjects taking aspirin or serotonin reuptake inhibitors were excluded from the study.

**Table 1:** Demographic and clinical characteristics of patients with SSc and control subjects

Characteristic	Patients (n = 30)	Control subjects (n = 12)
Female sex, %	96.6	91.6
Age, yr, mean (SD)	51.3 (16.8)	41.9 (14.7)
Disease duration, mo, mean (SD)	128.5 (18.4)	
Disease subset		
Diffuse systemic sclerosis, %	17.2	
Limited systemic sclerosis, %	82.8	
ANA positivity (>1:80), %	100	0
Centromere, %	71.4	
Nucleolar, %	3.7	
Other, %	25	
Anti-Scl-70	16.1	
Anti-RNA polymerase III	6.7	
ESR, mm/h, mean (SD)	32.7 (33.6)	
Hemoglobin, g/dl, mean (SD)	12.5 (1.2)	
WBC, n/μl, mean (SD)	7288.21 (2836.6)	
Platelets, 10^9^/L, mean (SD)	245 (65)	
CRP, mg/L, mean (SD)	0.47 (0.5)	
Ultrasensitive CRP μg/ml, mean (SD)	12.24 (28.2)	5.08 (1.0)
Complement component C3, mg/dl, mean (SD)	104.42 (20.2)	
Complement component C4, mg/dl, mean (SD)	17.42 (5.79)	
Raynaud’s phenomenon, %	89.66	
Gastrointestinal symptoms, %	75.0	
Digital ulcers, %	31.3	
Calcinosis, %	17.2	
Lung fibrosis, %	23.3	
Pulmonary arterial hypertension, %	13.3	
Current therapies, %		
Mycophenolate mofetil	16.7	
Methotrexate	26.7	
Cyclophosphamide	6.7	
Prednisone	18.7	

*ANA* antinuclear antibodies, *CRP* C-reactive protein, *ESR* erythrocyte sedimentation rate, *IQR* interquartile range, *SD* standard deviation, *WBC* white blood cell count

### Platelet isolation

Platelets were prepared as previously described [13]. Briefly, venous blood (40 ml) was collected from patients and control subjects and diluted in acid citrate dextrose formula A solution (1:10 vol/vol). After centrifugation (10 minutes at 150 × *g*) the platelet-rich plasma was collected and centrifuged (10 minutes at 150 × *g*). The pellet was washed with Tyrode’s solution (137 mM NaCl, 5.3 mM KCl, 1 mM MgCl_2_, 2 mM CaCl_2_, 4.1 mM NaCO_3_, and 5.5 mM glucose, pH 6.5, containing 120 nM prostaglandin E_1_). Platelets were centrifuged to remove residual leukocytes and finally resuspended in the same Ca^2+^-free buffer. Leukocyte contamination was evaluated by fluorescence microscopy using propidium iodide staining. Leukocyte counts were always less than 1/10^6^ platelets. In addition, monocyte contamination was evaluated by amplification of CD14 messenger RNA (mRNA) in polymerase chain reactions (PCRs). Isolated, washed platelets at 600,000/μl were incubated in the presence or absence of 2 μg/ml equine collagen (collagen reagent; Hormon-Chemie, Munich, Germany) for 5 minutes at 37 °C in a four-channel PAP-4 aggregometer (Bio/Data, Horsham, PA, USA). The slope and maximal platelet aggregation were recorded for 5 minutes. Then platelet suspensions were centrifuged at 10,000 × *g* for 5 minutes, and supernatants were collected and stored at −80 °C until processing.

### Measurement of inflammatory mediators

Transforming growth factor β (TGF-β), CD40L, tumor necrosis factor (TNF)-α, VEGF, and VEGF_165_b were measured in platelet supernatants by using commercial quantitative colorimetric sandwich enzyme-linked immunosorbent assays (ELISAs) (catalogue numbers DB100B, DCDL40, DTA00C, DVE00, and DY3045, respectively; R&D Systems, Minneapolis, MN, USA) and connective tissue growth factor (CTGF) was measured in platelet supernatants by using another ELISA (catalogue number RHF461CKX; Antigenix America, Huntington Station, NY, USA) according to the manufacturers’ instructions. Concentrations were calculated using a standard curve generated with specific standards provided by the manufacturers. Samples for TGF-β analysis were acid-activated with 1 N HCl. Optical density was measured with a microtiter plate reader at 450 nm. Each sample was measured in triplicate. von Willebrand factor (VWF) was measured in serum by ELISA as described previously [14].

### Intraplatelet serotonin determination

Intraplatelet serotonin (5-hydroxytryptamine) was measured using a high-performance liquid chromatography (HPLC) technique [15]. The HPLC system consisted of Ultrasphere 5-μm ODS column, 250 × 4.6 mm (HiChrom, Theale, UK), a Waters 515 HPLC pump (Waters, Milford, MA, USA), a Rheodyne manual injector (Sigma-Aldrich, St. Louis, MO, USA), an electrochemical detector (Waters 464), and EMPOWER software (Waters). A platelet sample (20 μl) was injected for HPLC analysis, and the amount of serotonin was calculated on the basis of a calibration curve.

### Angiogenesis and fibroblast proliferation assays

To assess platelet-derived angiogenic and antiangiogenic factors, in vitro tubule formation assays were performed with human DMVECs [catalogue number CRL-4025; American Type Culture Collection (ATCC), Manassas, VA, USA] cultured in 24-well plates covered with Matrigel culture mix (BD Biosciences, San Diego, CA, USA). Briefly, Matrigel (200 μl) was pipetted into culture wells and polymerized for 30 minutes at 37 °C. Then DMVECs that formed a small number of short tubular structures when cultured alone were cultured in duplicate in endothelial growth basal medium (EBM-2) supplemented with EBM-2MV SingleQuots (Lonza, Walkersville, MD, USA) on 24-well plates. Each experiment was conducted by pairing samples of platelet supernatants (10 % vol/vol) derived from patients with SSc and from healthy control subjects. As a control, DMVECs were supplemented with VEGF (10 ng/ml) or cultured alone on Matrigel. Cells were photographed at 6 h. The results were quantified by measuring the total tube length in each well.

To assess platelet-derived TGF-β, human lung fibroblasts (WI-38, catalogue number CCL-75; ATCC) were cultured in 24-well plates in Dulbecco’s modified Eagle’s medium (DMEM) supplemented with 10 % fetal bovine serum. For proliferation assays, fibroblasts were cultured with 10 % vol/vol SSc or control platelet supernatant and after 36 h and proliferation was assessed with the CellTiter 96 Non-Radioactive Cell Proliferation Assay [3-(4,5-dimethylthiazol-2-yl)-2,5-diphenyltetrazolium bromide (MTT); Promega, Madison, WI, USA) according to the manufacturer’s instructions. Briefly, after 36 h in fibroblast culture, MTT was added to the cells, followed by incubation at 37 °C for 4 h. Then each well was incubated for 1 h with solubilization stop solution, and absorbance was analyzed using an ELISA reader at 570 nm. Untreated fibroblasts were used as a negative control. The results were normalized to untreated cells. Fibroblasts incubated with 5 ng/ml TGF-β were used as a positive control.

### Quantitative real-time RT-PCR of endothelin-1 and α-smooth muscle actin

Gene expression was quantified by performing TaqMan real-time reverse transcription (RT)-PCR with a PRISM 7500 System (Applied Biosystems, Foster City, CA, USA). Total RNA was isolated from DMVECs and fibroblasts with TRIzol reagent (Life Technologies, Grand Island, NY, USA). RT into complementary DNA (cDNA) was performed using avian myeloblastosis virus reverse transcriptase (Promega), with random primers. A predeveloped *RPL32* TaqMan gene expression assay (Hs00851655-g1; Life Technologies) was used to normalize for the amounts of loaded cDNA. Differences were calculated with the comparative cycle threshold method for relative quantification. Endothelin-1 (Hs00174961-m1; Life Technologies) and α-smooth muscle actin (α-SMA) (Hs00426835-g1; Life Technologies) gene expression was evaluated with TaqMan assays.

### Statistical analyses

Statistical analyses were performed using SAS version 9.1 software (SAS Institute, Cary, NC, USA). Data are shown as mean ± standard error of the mean or median and range. A two-sided non-parametric Mann–Whitney *U* test was used for independent samples, and Student’s *t* test was performed for paired samples. Analysis of variance (ANOVA) was used for multiple comparisons. *p* < 0.05 was considered statistically significant.

## Results

### DMVEC angiogenesis is inhibited by SSc platelet releasates

**Fig. 1:**
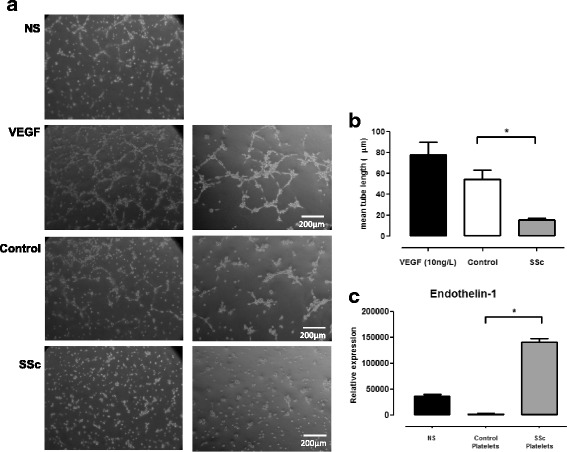
Systemic sclerosis (SSc) platelet releasate inhibits angiogenesis. **a** Representative images of Matrigel cultures of dermal human microvascular endothelial cells (DMVECs) after 6 h in culture. Healthy control and SSc platelet supernatants were cultured at 10 % vol/vol. *Left* and *right columns* represent different magnifications (magnification is 10x for the first column and 20x for the second column). **b** Capillary morphogenesis was quantified by measuring the length of capillary projections (*p < 0.01, *t* test). Data are mean ± standard deviation of six duplicate experiments. **c** Endothelin-1 mRNA expression in DMVECs after 24 h of culture in platelet-conditioned medium. Endothelin-1 was upregulated after the cells were cultured with SSc platelet supernatants (*p* < 0.05, Mann–Whitney *U* test). *NS* non-stimulated, *VEGF* vascular endothelial growth factor

Tissue fibrosis is preceded by microvascular injury in SSc, leading to a progressive loss of capillaries [16]. The effect of platelet releasates in angiogenesis was studied using a tubule formation assay with DMVECs cultured in Matrigel for 6 h. The capacity of platelet releasates to enhance tubular formation was expressed as the total tube length in the culture. Figure 1a–b shows that the conditioned medium from SSc platelets significantly inhibited tubule formation in Matrigel cultures compared with supernatants from healthy control platelets. The graph shows the results of six different duplicate experiments (*p* < 0.01, *t* test). We found no differences according to disease subset (data not shown).

We also evaluated endothelin-1 mRNA expression in DMVECs after 24 h of culture in medium (control) or platelet releasates from healthy control subjects and patients with SSc. Endothelin-1 was upregulated after culturing the cells for 6 h with SSc platelet supernatant (Fig. 1c) (*p* < 0.05, Mann–Whitney *U* test for healthy control vs. SSc platelet releasates). Moreover, the supernatants from healthy control platelets reduced the endothelin-1 mRNA expression compared with non-stimulated DMVECs.

**Fig. 2:**
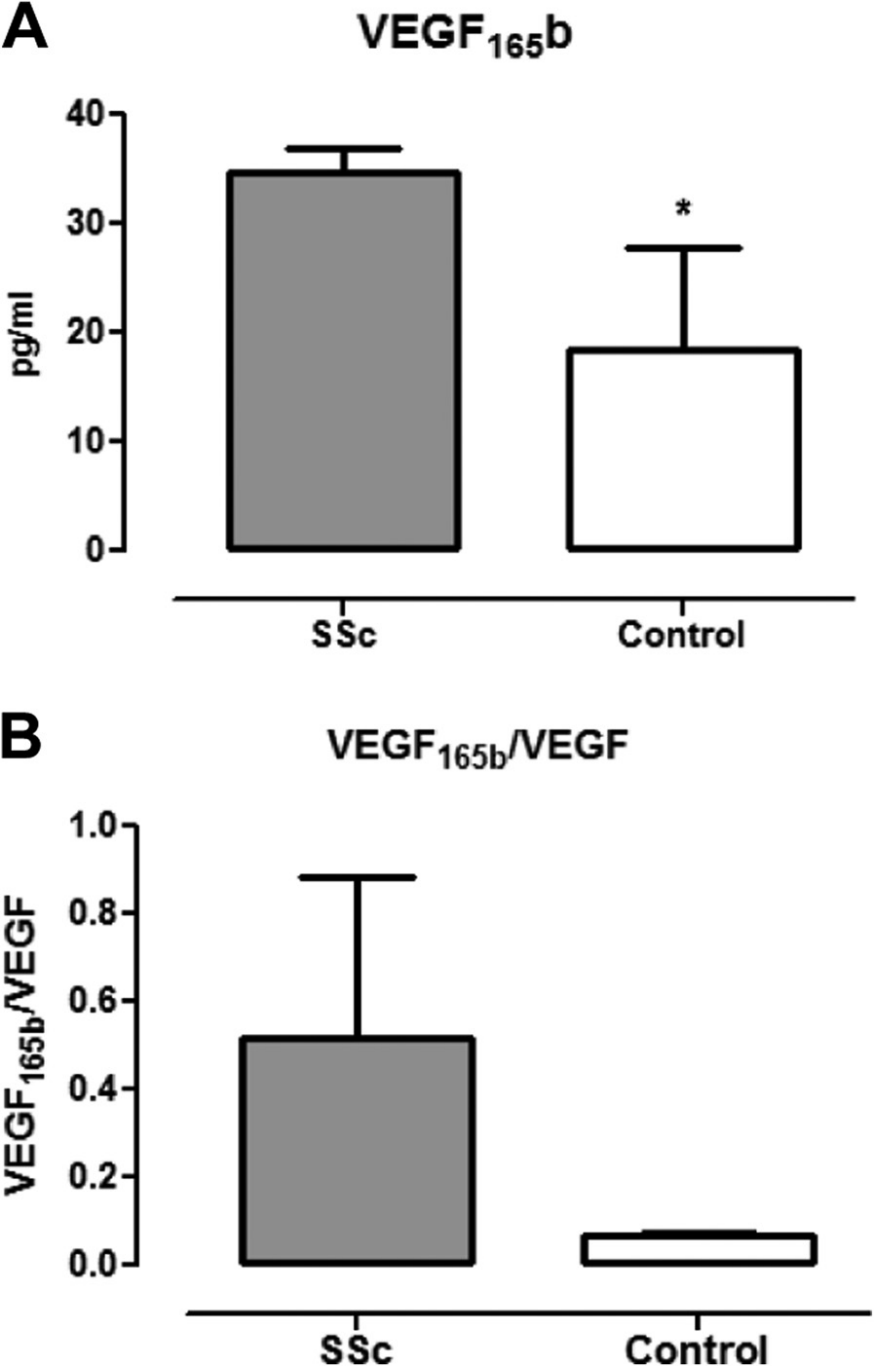
Antiangiogenic factors predominate in systemic sclerosis (SSc) platelet releasates. **a** Vascular endothelial growth factor, 165b isoform (VEGF_165_b), determination in platelet supernatants. We observed significantly higher levels of VEGF_165_b in SSc platelet supernatants (*p < 0.05, *t* test). **b** Total vascular endothelial growth factor (VEGF) was evaluated in platelet supernatants, and a relationship between VEGF_165_b and VEGF was established. SSc platelets releasate showed increased levels of the antiangiogenic factor VEGF_165_b

Several studies have shown that VEGF expression is markedly increased in the epidermis and dermis of patients with SSc [17, 18]. As recently reported [19], there are splice variants of VEGF with antiangiogenic effects, one of them being VEGF_165_b. Increased VEGF_165_b has been shown in serum and in skin biopsies of patients with SSc [20]. Given that platelets contain high amounts of VEGF, we measured the levels of VEGF_165_b in platelet supernatants. As shown in Fig. 2a, SSc platelet supernatants contained significantly higher levels of VEGF_165_b than healthy control platelet supernatants (*n* = 12; *p* < 0.05, *t* test). The ratio of antiangiogenic VEGF_165_b to total VEGF in platelet supernatants was higher in patients with SSc than in control subjects, but the difference did not reach statistical significance (*n* = 12; *p* = 0.071, *t* test) (Fig. 2b). Taken together, these results support an antiangiogenic role of VEGF_165_b in microvascular damage.

### Proinflammatory and profibrotic cytokines secreted by SSc-derived platelets

**Fig. 3:**
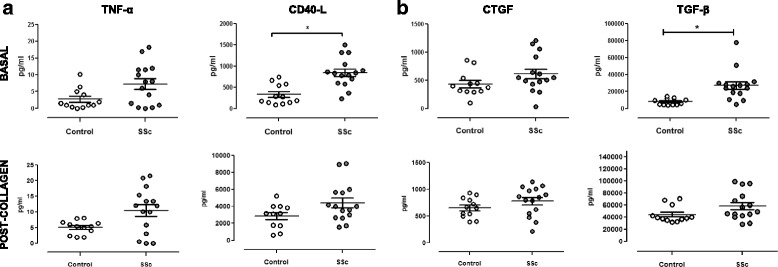
Systemic sclerosis (SSc) platelets secrete higher amounts of tumor necrosis factor (TNF)-α, CD40L, connective tissue growth factor (CTGF), and transforming growth factor (TGF)-β cytokines. Basal and postcollagen proinflammatory cytokines TNF-α (**a**) and CD40L (**b**) profibrotic cytokines CTGF and TGF-β were determined in platelet supernatants of patients with SSc and healthy control subjects. We observed higher levels of CD40L and TGF-β in SSc platelets before collagen activation. *p < 0.01, *t* test

The releasates (basal and postcollagen) obtained after platelet isolation were analyzed for TGF-β, TNF-α, CTGF, and CD40L levels (Fig. 3a, b). We observed significant increases in basal TGF-β (*p* < 0.01, *t* test) and CD40L (*p* < 0.01, *t* test) secretion in platelets from patients with SSc compared with those from healthy control subjects. After collagen stimulation, platelets from patients with SSc also secreted higher levels of TGF-β and CD40L, although the differences were non-significant in this experimental setting (*p* = 0.07 for TGF-β and *p* = 0.05 for CD40L, *t* tests). The slight increases of TNF-α and CTGF in basal and activated supernatants of SSc platelets compared with supernatants of control platelets were not statistically significant.

**Fig. 4:**
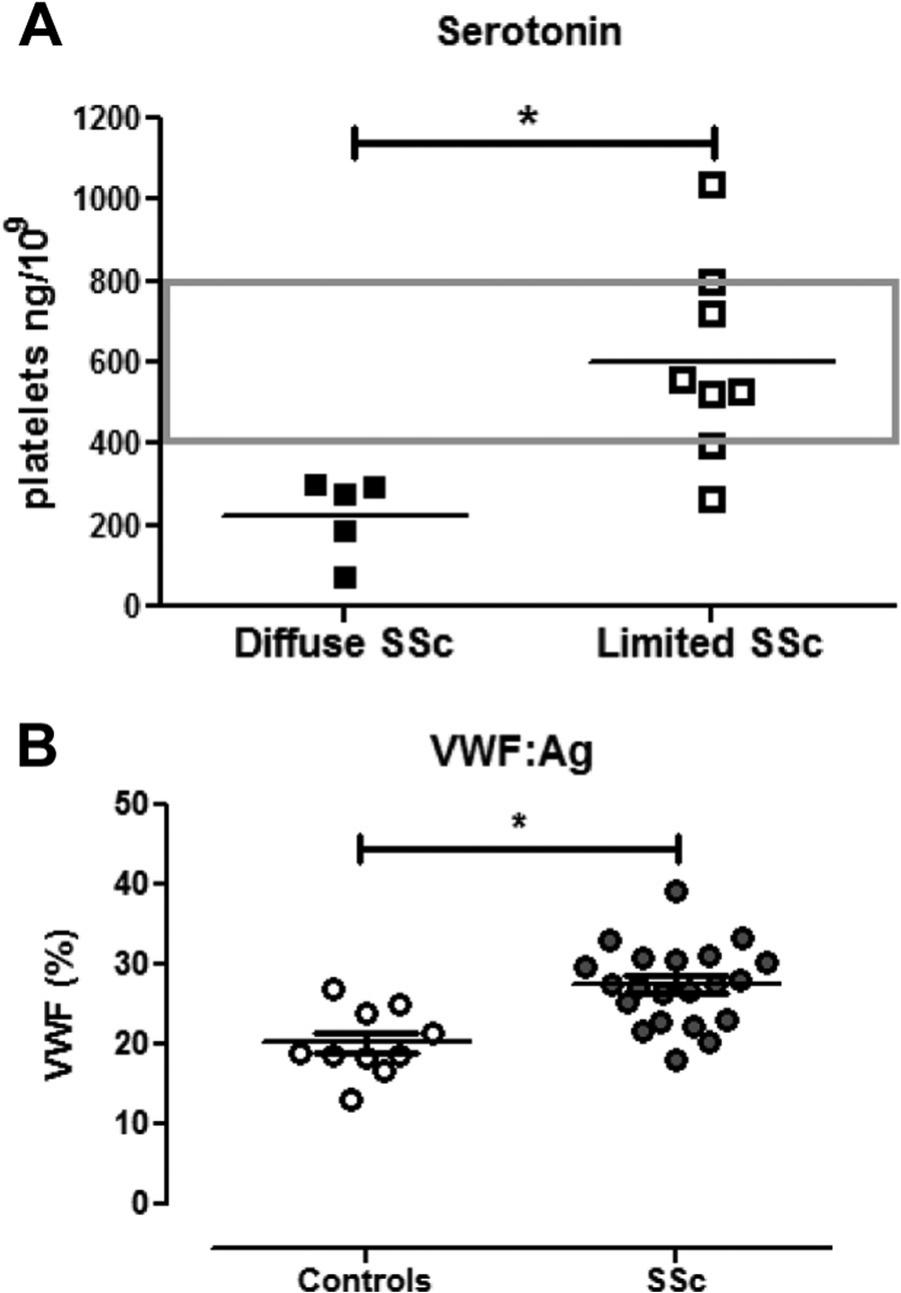
**a** Intraplatelet serotonin levels are decreased in patients with diffuse systemic sclerosis (SSc) compared with patients with limited SSc (*p < 0.05, *t* test). **b** von Willebrand factor antigen (VWF:Ag) was evaluated in serum of patients with SSc and healthy control subjects. Higher levels are observed in patients with SSc (*p < 0.05, *t* test)

It has been suggested that platelet-derived serotonin can stimulate extracellular matrix synthesis in interstitial fibroblasts [21]. This observation led us to measure intraplatelet serotonin content in patients with SSc. As observed in Fig. 4a, serotonin is significantly reduced in patients with diffuse SSc compared with patients with limited SSc and healthy control subjects (*p* < 0.05, t test). We also observed increased levels of VWF, a biomarker of endothelial activation, in the serum of patients with SSc (Fig. 4b).

### Fibroblast proliferation and α-smooth muscle actin expression

**Fig. 5:**
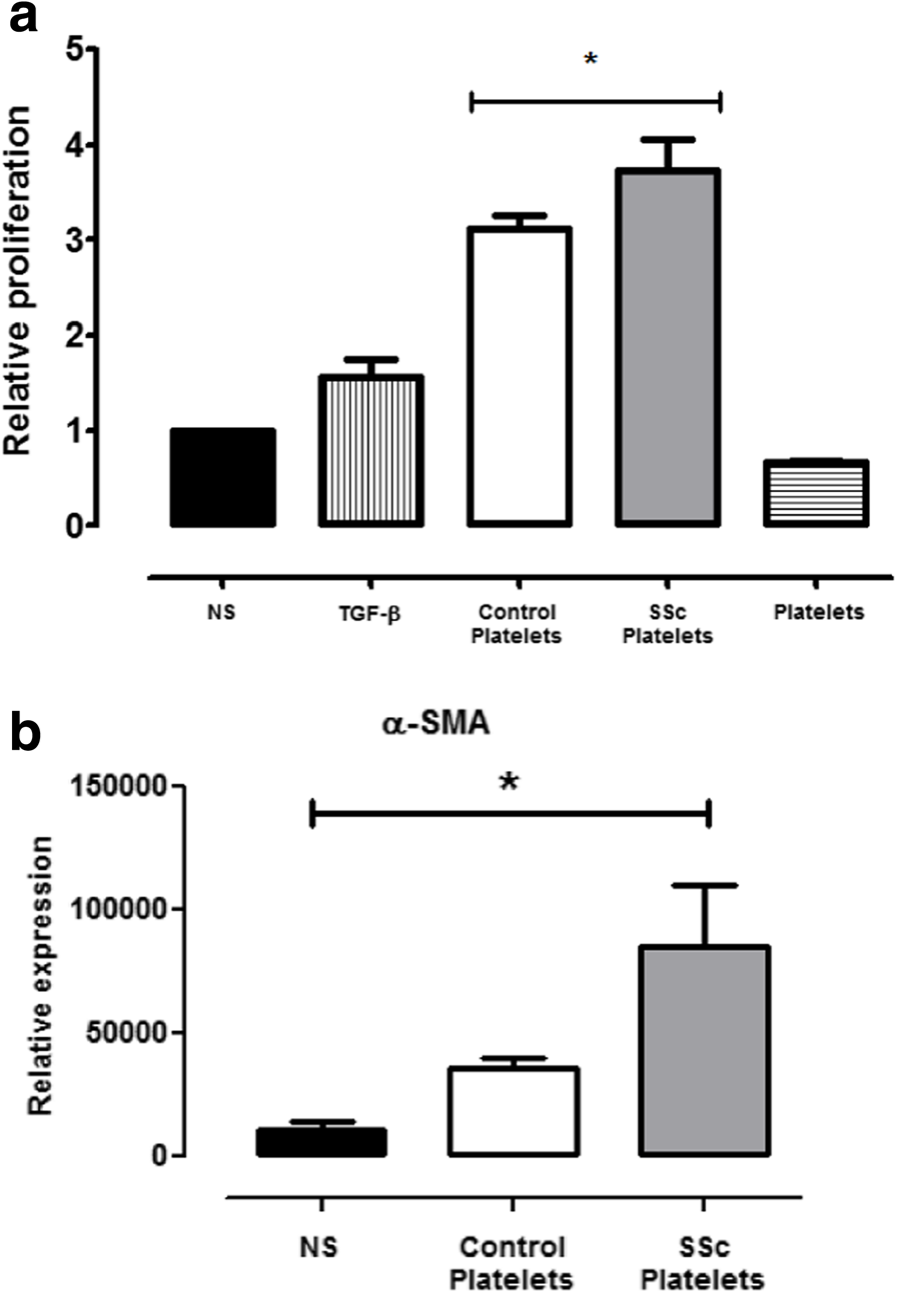
Systemic sclerosis (SSc) platelet supernatant stimulates fibroblast proliferation and α-smooth muscle actin (α-SMA) expression. **a** Human fibroblast proliferation was evaluated using a 3-(4,5-dimethylthiazol-2-yl)-2,5-diphenyltetrazolium bromide assay after 24 h culture alone [non-stimulated (NS)] or with transforming growth factor (TGF)-β as well as with platelet releasates from patients with SSc and from healthy control subjects. The *rightmost bar* is a negative control and corresponds to normal platelets. Higher proliferation was observed after incubation with SSc platelet supernatants than with healthy control supernatants (*p < 0.05, Mann–Whitney *U* test). **b** In the same experiment, α-SMA expression was evaluated in fibroblasts. Higher α-SMA expression was found in fibroblasts incubated with SSc platelet releasates than with healthy control releasates (*p < 0.05, analysis of variance). Expression was determined by performing real-time polymerase chain reactions using TaqMan assays

Tissue fibrosis in SSc results from an increased release of extracellular matrix from aberrantly activated fibroblasts [16]. Therefore, we evaluated the effect of platelet releasates in human fibroblast proliferation and α-SMA expression. Human lung fibroblasts were cultured in 24-well plates in DMEM supplemented with 10 % vol/vol SSc or healthy control platelet supernatant. Figure 5a shows that SSc platelet releasate supernatants induced stronger fibroblast proliferation than healthy control platelet releasate supernatants (*p* < 0.001, ANOVA; *p* < 0.05, *t* test; control vs. SSc platelet groups). Also, we observed greater α-SMA expression by fibroblasts cultured with SSc than by control platelet releasates (*p* < 0.05, ANOVA).

## Discussion

Platelets are best known for their role in hemostasis and thrombosis, but they also play important roles in inflammation, angiogenesis, and wound healing. Due to their small size and permanent circulation, they are in close proximity to and in continuous interaction with the endothelium. The sprouting of new vessels and the activation of fibroblasts stimulated by platelets within injured tissues probably contribute to prevention of hemorrhage and accelerated wound healing. However, when this remodeling response occurs with no regulation or in the absence of an injury stimulus, the integrity of the vessel and the tissue surrounding it can be impaired. Some of the first evidence that platelets could play a role in SSc pathogenesis was reported by Kahaleh et al. [8], who showed elevated levels of circulating platelet aggregates and higher levels of β-thrombomodulin in patients with SSc.

Microvascular damage is an early event in scleroderma. It involves small vessels, particularly arterioles. Immunohistochemical studies of skin biopsy samples at different disease stages indicate that the initial injury affects endothelial cells [22, 23] with loss of integrity of the endothelial lining, where gaps between cells and vacuolization of endothelial cell cytoplasm have been described [24]. These endothelial alterations lead to microvascular dysfunction, which may precede by many years the appearance symptoms and involvement of internal organs, when fibrosis becomes evident [25]. Endothelial injury results in platelet activation and release of cytokines. In this study, we observed that platelets from patients with SSc, compared with healthy control platelets, inhibited angiogenesis in a microtubule formation assay using DMVECs. This angiostatic effect may be a contributing factor to microangiopathy in patients with SSc. Consistent with this observation, we detected increased expression of endothelin-1 in endothelial cells incubated with SSc platelet supernatants [26] and inhibition when the same cells were incubated with releasates from normal platelets. We cannot be certain which platelet releasate component might contribute to these contrasting observations. Interestingly, the authors of a recent report found that serum containing anti–endothelial cell antibodies from patients with SSc induced endothelin-1 secretion from microvascular endothelial cells [27]. However, we did not test anti–endothelial cell antibodies in our patients.

Several studies have shown that the expression of VEGF, a major proangiogenic regulator, is increased in different cell types in the epidermis and dermis of these patients [18] and that the circulating levels of VEGF are also significantly increased in patients with SSc [17, 28]. However, in SSc, angiogenesis is defective and the disease evolves toward a progressive loss of capillary vessels. Interestingly, the antiangiogenic splice variant VEGF_165_b is upregulated in SSc skin and circulation [20, 29], and it was suggested that profibrotic TGF-β may contribute to the switch from proangiogenic to antiangiogenic VEGF isoforms [19]. The higher concentration of VEGF_165_b in SSc platelet releasates that we observed, which is consistent with those previous reports, may contribute to angiogenesis inhibition and microangiopathy. It is well known that, among the more than 1000 proteins demonstrated in platelet releasates [30, 31], platelets contain several angiogenesis regulatory proteins in their α-granules. Angiogenesis is regulated by a highly sensitive interplay of growth factors and inhibitors, and their imbalance may lead to disease. In this regard, the increased ratio of antiangiogenic to angiogenic VEGF isoforms observed in these patients could be explained by increases in the antiangiogenic splice variant VEGF_165_b. Furthermore, researchers in a recent study found that the levels of C-X-C motif chemokine ligand 4/platelet factor 4, which is platelet-specific, and antiangiogenic and profibrotic chemokines are increased in patients with SSc and correlate with the level of fibrosis as well as the occurrence and progression of pulmonary arterial hypertension [32].

Some of the assessed platelet cytokines were increased in SSc. CD40L, a transmembrane protein of the TNF family, is expressed not only by immune system cells but also by activated platelets [33]. It is stored in platelets and is externalized and secreted during platelet activation [34, 35]. We observed that CD40L secretion in platelets from patients with SSc was significantly increased compared with platelets of control subjects (*p* < 0.01, *t* test). We also found that TGF-β, a growth factor released from activated platelets, was significantly higher in platelet releasates from patients than in those from control subjects. TGF-β stimulates angiogenesis, smooth muscle cells, and connective tissue fibroblasts [36], promoting the synthesis of collagens and matrix components [37]. In this regard, platelets are the main source of these molecules in the circulation [38–40], and the authors of previous reports have described increased levels of serum CD40L in scleroderma patients [41] and increased serum TGF-β in patients with Raynaud’s phenomenon [10].

Platelets contain almost all of the circulating serotonin, a vasoactive monoamine that is released upon platelet activation and which has been found to be increased in the plasma of patients with SSc [42]. We observed that intraplatelet serotonin was decreased in patients with diffuse SSc compared with patients with limited SSc and healthy control subjects. Given that the serotonin content of platelets increases as platelets age in the circulation, this finding may be explained by a decreased platelet lifespan in the circulation with a consequent increased turnover in diffuse SSc [43]. However, this is also compatible with the notion of damaged endothelium-mediated platelet activation and accelerated removal of platelets from the circulation. In this regard, TGF-β and serotonin have been observed to be involved in fibrosis generation [21].

Normally, under the influence of appropriate stimuli, fibroblasts or their progenitor cells synthesize collagens and other extracellular matrix glycoproteins; secrete growth factors, cytokines, and chemokines; express surface receptors for these ligands; and undergo transdifferentiation into myofibroblasts [44]. These are specialized cells that arise from fibroblasts in response to TGF-β stimuli, which express the cytoskeletal protein α-SMA and synthesize collagens, tissue inhibitors of metalloproteases, and other extracellular matrix components [45, 46]. Several alterations in fibroblasts have been described in the pathogenesis of scleroderma [25]. Our results show that platelet releasates from patients with SSc stimulate human fibroblast proliferation and increase α-SMA expression, further contributing to disease progression.

## Conclusions

Platelets are involved in several forms of pathologic angiogenesis, tissue repair, and fibrosis. From this point of view, our results highlight the role of platelets in SSc pathogenesis. Our findings suggest that antiangiogenic factors such as VEGF_165_b, together with proinflammatory (CD40L) and profibrotic (TGF-β) factors secreted by platelets, can contribute to the defective angiogenesis and vascular repair in SSc. A better understanding of how platelets transport and deliver angiogenic, inflammatory and fibrotic regulatory proteins, as well as their contribution to SSc development and perpetuation, may have novel therapeutic implications.

## Abbreviations

ANA: antinuclear antibodies
ANOVA: analysis of variance
ATCC: American Type Culture Collection
cDNA: complementary DNA
CRP: C-reactive protein
CTGF: connective tissue growth factor
DMEM: Dulbecco’s modified Eagle’s medium
DMVEC: dermal human microvascular endothelial cell
EBM-2: endothelial growth basal medium
ELISA: enzyme-linked immunosorbent assay
ESR: erythrocyte sedimentation rate
HPLC: high-performance liquid chromatography
IQR: interquartile range
mRNA: messenger RNA
MTT: 3-(4,5-dimethylthiazol-2-yl)-2,5-diphenyltetrazolium bromide
NS: non-stimulated
PCR: polymerase chain reaction
RT: Reverse transcription
SD: standard deviation
α-SMA: α-smooth muscle actin
SSc: systemic sclerosis
TGF-β: transforming growth factor β
TNF-α: tumor necrosis factor α
VEGF: vascular endothelial growth factor
VEGF_165_b: vascular endothelial growth factor, 165b isoform
VWF: von Willebrand factor
WBC: white blood cell count
